# Southeast Asian Medicinal Plants as a Potential Source of Antituberculosis Agent

**DOI:** 10.1155/2017/7185649

**Published:** 2017-07-03

**Authors:** Shuaibu Babaji Sanusi, Mohd Fadzelly Abu Bakar, Maryati Mohamed, Siti Fatimah Sabran, Muhammad Murtala Mainasara

**Affiliations:** Centre of Research for Sustainable Uses of Natural Resources (CoR-SUNR), Faculty of Science, Technology and Human Development, Universiti Tun Hussein Onn Malaysia (UTHM), 86400 Parit Raja, Batu Pahat, Johor, Malaysia

## Abstract

Despite all of the control strategies, tuberculosis (TB) is still a major cause of death globally and one-third of the world's population is infected with TB. The drugs used for TB treatment have drawbacks of causing adverse side effects and emergence of resistance strains. Plant-derived medicines have since been used in traditional medical system for the treatment of numerous ailments worldwide. There were nine major review publications on antimycobacteria from plants in the last 17 years. However, none is focused on Southeast Asian medicinal plants. Hence, this review is aimed at highlighting the medicinal plants of Southeast Asian origin evaluated for anti-TB. This review is based on literatures published in various electronic database. A total of 132 plants species representing 45 families and 107 genera were reviewed; 27 species representing 20.5% exhibited most significant* in vitro* anti-TB activity (crude extracts and/or bioactive compounds 0–<10 *µ*g/ml). The findings may motivate various scientists to undertake the project that may result in the development of crude extract that will be consumed as complementary or alternative TB drug or as potential bioactive compounds for the development of novel anti-TB drug.

## 1. Introduction

Tuberculosis (TB) is an ancient disease and it is among the world's most deadly epidemics. Like any other infectious disease, TB can happen to anyone and spares no age, sex, and nationality [1, 2]. Several strains of* Mycobacterium tuberculosis* (MTB) are the common cause of this deadly infectious disease [3]. This disease is endemic in every country in the world, and death due to TB is more common when compared to other bacterial disease [1, 3, 4]. About two billion individuals are latently infected with TB, but only 10% of these infected persons fall sick with active disease during their lifetime [5–8]. It has been estimated that around nine million persons develop TB and almost 2 million die from it annually [9–12]. About 5.5 million of the cases occur in Asia, 1.5 million in Africa, 745,000 in the Middle East, and 600,000 in Latin America [13]. It is unfortunate that more than 75% of TB cases are found in adults [14]. Unprecedented decision was taken in 1993 by WHO to declare TB as a public health emergency [10, 15], and it is the first disease that has ever been declared as a global emergency by WHO [16].

In order to combat TB, chemotherapy is used, which is the modern TB treatment. The drugs used include rifampicin, isoniazid, ethambutol, pyrazinamide, and streptomycin for TB treatment. However, these drugs have drawbacks of causing adverse side effects and the TB-causing bacterium can gain easy resistance against these drugs. Besides that, there is a chance of “relapse” due to noncompliance with medication within the first year of treatment. Consequently, this results in a more serious condition where the* Mycobacterium* species develops resistance to the TB drugs [1].

The TB resistance can be categorized into two types: the multi-drug resistant TB (MDR-TB) and extensively drug resistant TB (XDR-TB). Firstly, MDR-TB arises when the strain that is resistant to the first-line standard TB drugs (isoniazid and rifampicin) is involved. More than 4% of TB patients globally are infected with* Mycobacterium* strains that are resistant to first-line drugs. Secondly, XDR-TB happens when the mycobacterial strain is resistant to the first-line drugs, fluoroquinolone, as well as other injectable second-line drugs such as kanamycin, capreomycin, and amikacin. According to the WHO report on surveillance and response to MDR-TB and XDR-TB, approximately 310,000 MDR-TB cases occurred in pulmonary TB patients documented in 2011 while 84 countries were recorded having at least one XDR-TB case [17–20]. In 2012, around 480,000 people have been reported to develop MDR-TB and about 170,000 people died as a result [20, 21]. These forms of TB diseases are often more fatal, costly, and difficult to treat. The second-line drugs used in drug resistant TB have notable side effects, but have about 50% cure rate [22]. Although fluoroquinolones such as ofloxacin and norfloxacin have been used and are considered safer than the aforementioned second-line drugs, however, they also have their own drawback of being more costly.

Due to aforementioned disadvantages, the prospective efficacy of medicinal plants has motivated doctors and scientists to turn to folk medicines for treatment of various chronic diseases, including TB [22]. Hence, the urgent need arises towards the search of a component with a higher anti-TB activity, with easy availability and without side effects [1, 6, 23].

Owing to their chemical diversity and significant role in the drug sighting and development, medicinal plants proffer a great hope to overcome these needs. The medicinal plants have been comprehensively used either as crude extracts or pure materials. However, very few species of medicinal plants have been thoroughly explored for their medicinal properties [24–26]. The plant-derived medicines have been utilized in traditional medical system for the treatment of different illnesses worldwide. Around 75% of the global populace solely depends on medicinal plants for primary health care [27, 28]. Consequently, there is so much interest in plant medicine during the last few decades leading to numerous species of medicinal plant being investigated for their pharmacological activities.

In the last 17 years, there were nine major review publications on antimycobacteria from natural products. Newton et al. [29] published a review paper on natural compounds with antimycobacteria derived from plant source, describing their potency in crude extracts as well as pure compounds from 123 plant species [29]. In 2003, Copp [30] published a review article that covered a wide range of natural bioactive products, with reported antimycobacterial activity within a period of twelve years (i.e., from 1990 to 2002) [30]. In another review, Okunade et al. [31] discussed 88 natural products and their synthetic analogues, mainly from plant source, and fungi as well as some aquatic organisms that displayed substantial activity against* M. tuberculosis* and other mycobacterial species in* in vitro* bioassays [31]. In their review, Pauli et al. [32] offered cross-linkage to the literatures of bioactive pure compounds with anti-TB and summarized more current advances in mycobacteriology and natural compounds chemistry tools innovation as well as their prospective to influence the primary steps in TB drug discovery process [32]. A review by Jachak and Jain [33] described recent target-based natural compounds that displayed antimycobacterial action [33]. Gautam et al. [23] reviewed different species of plant from a vast array of families that have exhibited antimycobacterial activity. Gupta et al. [34], in their review article, identified sixty-four medicinal plants used by traditional people in the treatment of leprosy [34].

Some antitubercular plants from Ayurveda as well as foreign origin have been reviewed by Arya [35] to give a scientific account on usage of antitubercular plants. Consequently, various phytochemicals such as alkaloids, flavonoids, tannins, xanthones, triterpenes, and quinones were involved in antitubercular activity [35]. The most recent among all the reviews is by Chinsembu [20] in 2016. The review focused on antimycobacterial natural products derived from both endophytes as well as medicinal plants of Africa, Asia, Europe, South America, and Canada. Several plant species disclosed in the review demonstrated a putative anti-TB activity. Numerous antimycobacterial bioactive compounds have, as well, been isolated. They include 1-epicatechol, allicin, anthocyanidin, anthraquinone glycosides, arjunic acid, benzophenanthridine alkaloids, beta-sitosterol, crinine, decarine, ellagic acids, ellagitannin punicalagin, friedelin, galanthimine, gallic acid, glucopyranosides, hydroxybenzoic acids, iridoids, leucopelargonidol, neolignans, phenylpropanoids, taraxerol, and termilignan B. The chemicals may offer leads on new and more effective drugs to minimize the predicament TB and lessen the drug resistant strains as well [20].

All the above review articles hardly highlighted any medicinal plant of Southeast Asian source. Even though there is an increasing availability of modern medicine in the Southeast Asia region, the use of traditional medicine remains popular [36]. Due to the enormous diversity of its flora, Southeast Asian region has a great potential for the discovery of novel active compounds. The countries in the region such as Malaysia, Indonesia, Brunei, and Thailand have a long history of using medicinal plant that proffers substantial pharmaceutical prospects [37].

## 2. Methodology

Related Scientific studies published in journals, books, and reports were reviewed. Relevant literatures were searched in Google Scholar and various electronic databases including Science Direct, IEEE Xplore, Scopus, SciFinder, and MEDLINE using a specific search terms including “TB”, “medicinal plants”, “anti-TB”, “Malaysia OR Philippines OR Indonesia Singapore OR Thailand OR Brunei OR Cambodia OR Laos OR Myanmar (Burma) OR Vietnam”. This review discussed studies from year 2000 to 2016.

## 3. Bioassay Guidance for Evaluating the Activity of Antituberculosis

Bioassay-guided fractionation is the modern practice presently used in identifying the active compound(s) present in crude extract(s). Due to the fact that the procedure comprises alternating stages of biological screening and active compound fractionation, in the last 20 years, sensitivity of fractionation techniques of pure natural compound has dramatically improved due to the vast advancements in chromatography. Consequently, this creates new paths for both yet to be investigated materials and previously studied genera. Hence, the new paths were given access to unanticipated chemical varieties and novel biological products [32]. To provide effective direction in discovery program of drug from natural resources, the development of novel phytochemical approaches becomes crucial in a bioassay-directed drug discovery. Another step is bioassay which should be selected wisely in relation to the crucial terminus, that is, the antibiotic screening on virulent mycobacterial strain in vivo. Interestingly, three effective antimycobacterial products have been sequestered from* Dracaena angustifolia* using this method [23, 32, 38].

### 3.1. Target Organism

It is obvious that the ideal organism to be targeted in the effort to discover anti-TB is the actual etiologic agent,* Mycobacterium tuberculosis* (MTB). The prominent pathogenic strain, MTB H37Rv (ATCC 27294), has fairly represented drug sensitivity profile of most drug sensitive clinical isolated strains. In primary screening, employing MDR strains of MTB is not crucial due to the fact that they are never “superbugs” that have resistant capacity to various drugs by the virtue of a particular mechanisms, such as effusion pump in some bacteria. However, they are rather the consequence of peculiar step by step mutations to particular drugs. Hence, it is anticipated that they would be susceptible to any novel biologically active product, which does not attack the same site as TB drugs currently in use do [23, 32, 38]. Due to its virulence, the pathogenic strain of MTB must be processed or handled only in a laboratory with biosafety level 3 (BL-3) set-up. In the BL-3 laboratory, individual working is required to don on a protective gear. Most researchers opted to employ avirulent, fast growing, and saprophytic strain of* Mycobacterium*. Example of such is* M. smegmatis* (ATCC 607) [38]. Other avirulent substitutes to work with instead of virulent strains of MTB are the slow-growing strains such as* M. tuberculosis* H37Ra (ATCC 25177) and* M. bovis* BCG (ATCC 35743). The above species are closely related to pathogenic MTB H37Rv strain in their antimycobacterial susceptibility profile as well as genetic configuration. For this, strains require the employment of a class 2 biosafety cabinet when working with these organisms [23, 38].

### 3.2. *In Vitro* Bioassays for Anti-TB Screening

#### 3.2.1. Agar Diffusion

The conventional diffusion assays (well or disk) were applied in various antimicrobial evaluation of compounds from natural sources only to indicate the presence or absence of growth inhibition at unspecified concentrations gradient and hence are never quantifiable when evaluating crude materials or novel products. The sizes of the zones of inhibition can be only interpreted as indicative of either microbial sensitivity or resistance with well characterized antibiotics. This is because the size of inhibition zone depends on both the rate of diffusion of biologically active agent and the growth rate of the targeted organism [32]. Agar diffusion assays need to be avoided with* Mycobacteria*, since these organisms with high lipid content in their cell wall are usually more sensitive to compounds of less-polarity [38]. Therefore, the diffusion of such compounds will be very slow compared to polar compounds with the same molecular weight on the aqueous agar. This might consequently produce smaller inhibition zones. Furthermore, polar active compounds of low molecular weight could diffuse to equilibrium prior to appearance of colonies in slow-growing mycobacterial strain. And if, at the equilibrium, the concentration is below the MIC, the zone of inhibition will never appear [38].

#### 3.2.2. Micro and Macro Agar Dilution

When screening extracts with known concentrations, fractions in an agar enable the MIC value to be determined and its activity to be quantified. Except in some fastidious species, many mycobacterial strains such as MTB tend to produce colonies effectively on Middlebrook 7H10 or 7H11 agar supplemented with Oleic acid, Albumin, Dextrose, and Catalase (OADC). The sample to be tested is added to the semisolid media at final concentration of 1% v/v or subsequently either 100–200 *µ*l medium to 96-well microplates, 1.5 ml to 24-well microplates, 4 ml to 6-well microplates, or 20 ml into usual Petri dishes of 150 mm diameter. Following the hardening of the medium, the inoculum can then be dropped on the surface of the agar using a micro pipette. Some of the volumes of inoculum recommended are as follows: 1–5 *µ*l for 96-well plates, 10 *µ*l for 6- or 24-well plates, and 100 *µ*l for normal Petri dishes. The plates are then incubated at 37°C overnight, after which they should then be inverted for the remaining period of incubation. The major shortcoming with such a bioassay is that it requires a minimum of 18 days to produce a visible colony of* Mycobacteria* [32, 38].

#### 3.2.3. Microbroth Dilution

Evaluation of susceptibility (bioactivity) of natural products using microplates with 96 wells proffers an edge because it requires little sample, is cheap, and is high-throughput. The mycobacterial species are often cultured in Middlebrook 7H9 broth supplemented with glycerol (0.5%), casitone (0.1%), Tween-80 (0.05%), and ADC (10%). In many strains of* Mycobacteria*, the growth can be evaluated quantitatively by the broth medium turbidity. However, the proneness of clumps formation makes the assay very challenging [23]. Nevertheless, the utilization of indicator dye like Alamar blue renders this technique more sensitive and rapid. The results of this assay can be read visually, although the reduced form of the dye is quantifiable using calorimeter. This is done by measuring the absorbance at 570 nm and then subtracting absorbance at 600 nm. On the other hand, the second approach has been proven to be more sensitive. Microbroth dilution tests should also be carried out using either resazurin or tetrazolium dyes. Therefore, a high-throughput assay of anti-TB is possible through using a microplate, spectrophotometrically or fluorometrically. These are quantitative assays that could detect even partial inhibition, making it possible to determine the relative activity of fractions from crude extracts using different concentrations [32, 38].

## 4. Electron and Fluorescence Microscopy Studies

Electron and fluorescence microscopy have been used effectively to examine the morphological changes during the growth of microorganisms. In addition, they can also be employed in an attempt to locate the target of action of the test extract-treated mycobacterial samples [39]. The scanning electron microscope (SEM) provides a relatively easy technique of surface morphology study of microorganisms at high magnification with a resolution of around 15 to 20 nm under ideal conditions. One of the largely untapped potentials of this apparatus is the study of the morphological changes after bacterial exposure to antimicrobial agents [40].

The cell-wall-attacking characteristic of the test extracts is revealed in electron microscopy studies. Because the main part of the cell wall of* Mycobacteria* is comprised of lipids, it is assumed that the extracts must possess some effect on them. The target suspected is obviously mycolic acid (predominant lipid). It has been shown that a loss of acid fastness occurs when the cells of* Mycobacteria* are grown in the presence of antibiotics. The staining characteristics of this bacterial cell can be mainly attributed to the mycolic acids presence. Thus, when investigating the staining properties of cultures treated with extract, the culture is grown as for SEM studies. The auramine rhodamine dye is then used to stain the cells and visualized using a fluorescence microscope [39].

## 5. Genomics Studies and Proteomics Analysis

The systematic study of the whole set of cellular genetic material is called genomics. This will proffer enormous potential in both drug target and antigen discovery. Furthermore, it enhances novel antibacterial agents and vaccine development through DNA sequencing, as well as bioinformatics analysis. In TB study, it was first practiced on MTB H37Rv strain. Its bioinformatic investigation showed the attribution of accurate functions (~40% of the 4,000 genes). Once the functional information is available, it usually enables researchers to pinpoint a possible drug target on the basis of their proposed biological role or their resemblance to known bacterial drug targets [41]. In antibiotic drug discovery, expression of genome-based profiling may represent a useful tool for three applications: (i) target identification, (ii) antibiotics mechanism-of-action (MOA) studies, and (iii) new types of cell assays development for the purpose of drug screening [42].

Although many productive outcomes can be revealed in genomic studies, it is only the proteomic analysis that can obtain the exact cellular information [43]. The term proteomics denotes the proteins expressed by a genome. It addresses the protein, which is the final genomic product. The advantage of proteomics is in the overcoming of a major shortcoming of DNA chip technology. It has been proven to be vital in the novel antimycobacterial drug development [44]. With the aid of a technique called two-dimensional gel electrophoresis coupled with mass spectrometry (2DE-MS), about 263 proteins were identified in* M. bovis* BCG and MTB strains [45]. For protein patterns analysis, it is still the analytical technique available with the best resolution and has appeared as robust and efficient for rapid protein identification. It is assisted by the database of total genome sequence [46].

## 6. Southeast Asian Medicinal Plants with Anti-TB Activity

Despite the huge medicinal plant research efforts from Southeast Asian region, literature search showed that very little research work has been carried out on anti-TB plants and published by researchers from the region. Considering the abundant biodiversity and traditional ethnomedicinal knowledge in Southeast Asia, there is vast potential to institute a dedicated anti-TB screening programme. This review paper describes the Southeast Asian medicinal plants from a wide array of families that have been evaluated for anti-TB activity in the region so far.

They have been computed in a table form describing the plant species, families, part of plants and solvents used,* in vitro* activity, and ethnopharmacological uses (see Table 1). Interestingly, these plants species were found mentioned in various traditional medicines. Out of the 132 plants species (from 45 different families and 107 genera) discussed in this review, 114 species (87%) had reported role in the treatment of TB or TB-like symptoms in ethnomedicine (Table 1). More specifically, 24 species (18.2%) were reported for TB, 14 species (10.6%) for leprosy, and 76 species (57.6%) for TB-related diseases such as asthma, bronchitis, coughing, whooping cough, pulmonary infectious, fever, and chest diseases in ethnomedicine. It was found that crude extracts from 32 species representing 24.2% of all (132 species) plants demonstrated significant anti-TB activity in* in vitro* assay (MIC values ranging from 10 to 100 *µ*g/ml). These plant species are* Aegle marmelos* (L.) Correa,* Alpinia galanga* (L.) Sw.,* Alpinia purpurata* K. Schum.,* Alpinia zerumbet* (Pers.) B. L. Burtt & R. M. Sm.,* Annona reticulate* L.,* Artocarpus rigidus* Blume,* Boesenbergia pandurata* (Roxb.) Schltr.,* Clausena excavata* Burm. f.,* Clausena harmandiana* (Pierre) Guillaumin,* Croton kongensis* Gagnep.,* Eclipta prostrata* (L.) L.,* Eriosema chinense* Vogel,* Feroniella lucida* Swingle,* Glycosmis pentaphylla* (Retz.) DC.,* Gynura divaricata* (L.) DC.,* Haplophragma adenophyllum* (Wall. ex G. Don) Dop,* Heliotropium indicum* Linn.,* Marsypopetalum modestum* (Pierre) B. Xue,* Micromelum minutum* Wight & Arn.,* Morinda citrifolia* Linn,* Orthosiphon stamineus* Benth.,* Piper betle* L.,* Piper chaba* Hunter,* Piper nigrum* L.,* Piper sarmentosum* Roxb.,* Rollinia mucosa* (Jacq.) Baill.,* Solanum spirale* Roxb.,* Tinospora crispa* (L.) Hook. F. & Thomson,* Uvaria microcarpa* Champ. ex Benth.,* Uvaria rufa* Blume,* Vitex trifolia* L., and* Zingiber officinale* Roscoe.

Some bioactive compounds that were isolated from the reviewed medicinal plants exhibited good anti-TB activity (MIC values ranged between <1 and 50 *µ*g/ml). Active compound, Abruquinone B, from* Abrus precatorius* L. exhibited MIC of 12.5 *µ*g/ml. From* Aglaia erythrosperma* Pannell, ethyl eichlerianoate, eichlerialactone, and aglaialactone (all showing MIC of 25 *µ*g/ml) and cabraleadiol, cabraleahydroxylactone, cabralealactone, and flavagline (all showing MIC values of 50 *µ*g/ml) were obtained. 1′Acetoxychavicol acetate isolated from* Alpinia galanga* (L.) Sw. exhibited MIC value of 0.024 *µ*g/ml. Pimaric acid, 9*α*-13*α*-epidioxyabiet-8 (14)-en-18-oic acid, and 15-hydrooxydehydroabietic acid obtained from* Anisochilus harmandii* Doan ex Suddee & A. J. Paton all showed MIC value of 50 *µ*g/ml. Lakoochins A and B isolated from* Artocarpus lakoocha* Roxb. showed MICs of 12.5 and 50 *µ*g/ml, respectively. Flavonoid artonin F, chromone artorigidusin, xanthone artoindonesianin C, flavonoid cycloartobiloxanthone, and flavonoid 7-demethylartonol E isolated from* Artocarpus rigidus* Wall. showed MIC values of 6.25, 12.5, 12.5, 25, and 50 *µ*g/ml, respectively. Active compounds obtained from* Camchaya calcarea* Kitamura are isogoyazensolides (MIC, 1.5 *µ*g/ml), goyazensolides, lychnophorolides A, isocentratherin, isogoyazensolides and 5-epi-isocentratherin (with the same MIC value of 3.1 *µ*g/ml), lychnophorolides B, 1(10),E,4Z,11(13)-germacratriene-12,6-olide-15-oic acid, and caffeic acid methyl ester (MICs, 6.2, 50, and 25 *µ*g/ml resp.). Caseargrewiin A, Caseargrewiin B, Caseargrewiin D, rel-(2S,5R,6R,8S,9S,10R,18S,19R)-18,19-diacetoxy-18,19-epoxy-6-methoxy-2-(2-methylbutanoyloxy)cleroda-3,13(16),14-triene, and rel-(2S,5R,6R,8S,9S,10R,18S,19R)-18,19-diacetoxy-18,19-epoxy-6-hydroxy-2-(2-methylbutanoyloxy)cleroda-3,13(16),14-triene (all showing MIC value of 12.5 *µ*g/ml) and Caseargrewiin C (showing MIC of 25 *µ*g/ml) were isolated from* Casearia grewiifolia* Vent. Cabraleadiol, allo-aromadendrane-10*β*, 14-diol, cabraleahydroxylactone, cabralealactone, allo-aromadendrane-10*β*, 13, 14-triol (all displaying MICs of 50 *µ*g/ml), and eichlerialactone (MIC, 25 *µ*g/ml) were obtained from* Chisocheton penduliflorus* Planch. ex Hiern. Fluroclausine A and heptazoline isolated from* Clausena guillauminii* Tanaka all showed MIC of 25 *µ*g/ml. Dentatin, O-methylated clausenidin, and 3-methoxycarbonylcarbazole isolated from* Clausena excavata* Burm. f. all showed MIC value of 50 *µ*g/ml. 1-(2-Hydroxy-4-methoxyphenyl)-3-(4-hydroxy-3-methoxyphenyl)propane isolated from* Combretum griffithii* Van Heurck & Müll. Arg. showed MIC of 3.13 *µ*g/ml. Globiferin, cordiachrome B, cordiachrome C (showing MICs of 6.2, 12.5, and 1.5 *µ*g/ml resp.), alliodorin, elaeagin, cordiachromene (same MIC value of 12.5 *µ*g/ml), and cordiaquinol C (MIC, 25 *µ*g/ml) were isolated from* Cordia globifera* W. W. Smith. The bioactive compounds of* Croton kongensis* Gagnep.,* ent*-1*β*,7*α*,14*β*-triacetoxykaur-16-en-15-one (MICs, 0.78, 1.56, and 3.12–12.5 *µ*g/ml),* ent*-7*α*,18-dihydroxykaur-16-en-15-one (MIC, 1.56 *µ*g/ml), and* ent*-16(S)-18-acetoxy-7*α*-hydroxykaur-15-one exhibited MIC of 1.56 *µ*g/ml. Furthermore, ent-18-acetoxy-7*α*-hydroxykaur-16-en-15-one, ent-1*β*,14*β*-diacetoxy-7*α*-hydroxykaur-16-en-15-one, ent-1*β*-acetoxy-7*α*,14 *β*-dihydroxykaur-16-en-15-one, and ent-7*α*,14*β*-dihydroxykaur-16-en-15-one showed MIC values ranging from 3.12 to 6.25 *µ*g/ml. Other active compounds isolated from* Croton kongensis* are* ent*-8,9-*seco*-8,14-epoxy-7*α*-hydroxy-11*β*-acetoxy-16-kauren-9,15-dione (MIC, 6.25 *µ*g/ml),* ent*-8,9-*seco*-7*α*-hydroxy-11*β*-acetoxykaura-8(14),16-dien-9,15-dione (MIC, 6.25 *µ*g/ml), and* ent*-8,9-*seco*-7*α*,11*β*-diacetoxykaura-8(14),16-dien-9,15-dione (MIC, 25.0 *µ*g/ml). Flavanone, dalparvone, and dalparvinene isolated from* Dalbergia parviflora* Roxb. showed MIC values of 12.5, 50, and 50 *µ*g/ml respectively. 3*β*-hydroxy-21-O-acetyl-24-methylenecycloartane from* Dasymaschalon dasymaschalum* (Blume) I. M. Turner exhibited MIC of 50 *µ*g/ml. Bioactive compounds isolated from* Dendrolobium lanceolatum* (Dunn) Schindl., Flavanones 1, flavanones, flavan, and 4′-hydroxy-7,8-(2′′,2′′-dimethylpyran)flavan, exhibited MICs of 6.3, 12.5, 25, and 25 *µ*g/ml, respectively. Compounds 3,4-methylenedioxy-10-methoxy-7-oxo[2]benzopyrano[4,3-b]benzopyran, karanjachromene, pinnatin, 3-methoxy-(3′′,4′′-dihydro-3′′,4′′-diacetoxy)-2′′,2′′-dimethylpyrano-(7,8 : 5′′,6′′)-flavone, desmethoxy kanugin, lacheolatin B, 3,7-dimethoxyflavone, and pachycarin D (showing MIC values of 6.25, 12.5, 12.5, 25, 50, 50, 50, and 50 *µ*g/ml, resp.) were isolated from* Derris indica* L. Betulinic acid from* Diospyros decandra* Lour., showing MIC of 25 *µ*g/ml. From* Diospyros ehretioides* Wall. ex G. Don, palmarumycins JC2 (MIC, 6.25 *µ*g/ml) and isodiospyrol A (MIC, 50 *µ*g/ml) were isolated. Diospyrin, isolated from* Diospyros glandulosa* Lace, showed MIC value of 6.25 *µ*g/ml. Betulinaldehyde isolated from* Diospyros rhodocalyx* Kurz exhibited MIC value of 25 *µ*g/ml. Dehydrolupinifolinol (MIC, 12.5 *µ*g/ml), flemichin D (MIC, 12.5 *µ*g/ml), eriosemaone A (MIC, 12.5 *µ*g/ml), lupinifolin (MIC, 12.5 *µ*g/ml), Khonklonginol A (MIC, 25 *µ*g/ml), Khonklonginol H (MIC, 25 *µ*g/ml), lupinifolinol (MIC, 25 *µ*g/ml), and Khonklonginol B (MIC, 50 *µ*g/ml) were isolated from* Eriosema chinense* Vogel. From* Erythrina fusca* Lour., erythrisenegalone (50 *µ*g/ml MIC), lonchocarpol A (50 *µ*g/ml MIC), and lupinifolin (25 *µ*g/ml MIC) were isolated. Bioactive compounds erystagallin A, erycristagallin, 5-hydroxysophoranone, erysubin F (all showing MICs of 12.5 *µ*g/ml), and 1-methoxyerythrabyssin II (showing MICs of 50 *µ*g/ml) were obtained from* Erythrina subumbrans* Merr. (E)-((E)-3-(4-methoxyphenyl)allyl)3-(4-hydroxyphenyl)acrylate from* Etlingera pavieana* (Pierre ex Gagnep.) R. M. Sm., exhibited MICs of 50 *µ*g/ml. 3′-formyl-2′,4′-dihydroxy-6′-methoxychalcone, a bioactive compound from* Friesodielsia discolor* (Craib) D. Das, showed MICs of 6.25 *µ*g/ml. From* Garcinia mangostana* L., *α*-mangostin (MIC, 6.25 *µ*g/ml), *β*-mangostin (MIC, 6.25 *µ*g/ml), *γ*-mangostin (MIC, 25 *µ*g/ml), garcinone D (MIC, 25 *µ*g/ml), garcinone B (MIC, 6.25 *µ*g/ml), mangostanin (MIC, 25 *µ*g/ml), mangostenone A (MIC, 25 *µ*g/ml), tovophyllin B (MIC, 25 *µ*g/ml), demethylcalabaxanthone (MIC, 12.5 *µ*g/ml), and trapezifolixanthone (MIC, 12.5 *µ*g/ml) were identified as active compounds. Active compound Liriodenine isolated from* Goniothalamus gitingensis* Elmer exhibited MIC of 16 *µ*g/ml. Active compounds (+)-altholactone (MIC, 6.25 *µ*g/ml), howiininA (MIC, 6.25 *µ*g/ml), and (−)-nordicentrine (12.5 *µ*g/ml) were isolated from* Goniothalamus laoticus* (Finet & Gagnep.) Bân. Coronarin E and 16-Hydroxylabda-8(17),11,13-trien-15,16-olide isolated from* Hedychium ellipticum* Buch.-Ham. ex Sm. showed MICs of 12.5 and 6.25 *µ*g/ml, respectively. Caniojane isolated from* Jatropha integerrima* Jacq. demonstrated MIC of 25 *µ*g/ml. Bioactive compounds, sandaracopimaradien-1*α*-ol and 2*α*-acetoxysandaracopimaradien-1*α*-ol isolated from* Kaempferia marginata* Carey, showed MICs of 25 and 50 *µ*g/ml, respectively. From* Morinda citrifolia* Linn., campesta-6,22-dien-5*α*,8*α*-epidioxy-3*β*-ol (2.5 *µ*g/ml), (E)-phytol (32 *µ*g/ml), and stigmasterol (32 *µ*g/ml) were obtained. Active compounds 2,4-bis(2-phenylpropan-2-yl)phenol isolated from* Momordica charantia* L. showed 14 *µ*g/ml MIC. 1*α*,13*β*,14*α*-trihydroxy-3*β*,7*β*-dibenzoyloxy-9*β*,15*β*-diacetoxyjatropha-5,11E-diene and 1*α*,8*β*,9*β*,14*α*,15*β*-pentaacetoxy-3*β*-benzoyloxy-7-oxojatropha-5,12-diene isolated from* Pedilanthus tithymaloides* (L.) Poit. demonstrated 12.5 and 50 *µ*g/ml MIC. From* Piper sarmentosum* Roxb., pellitorine, 1-(3,4-methylenedioxyphenyl)-1E-tetradecene, guineensine, sarmentine, and brachyamide B showed MICs of 25, 25, 50, 50, and 50 *µ*g/ml, respectively. Bidebiline E (6.25 *µ*g/ml), octadeca-9,11,13-triynoic acid (6.25 *µ*g/ml), and *α*-humulene (6.25 *µ*g/ml) were isolated from* Polyalthia cerasoides* (Roxb.) Benth. ex Bedd. Debilisone B, Debilisone C, and Debilisone E isolated from* Polyalthia debilis* (Piere) Finet & ganep exhibited MIC values of 25, 12.5, and 25 *µ*g/ml, respectively. 1-Heneicosyl formate and 6*β*-hydroxy-10-O-acetylgenipin from* Premna odorata* Blanco and* Rothmannia wittii* (Craib) Bremek. showed 8 and 12.5 *µ*g/ml MIC values, respectively. The compounds chabamide and piperine isolated from* Piper chaba* Hunter exhibited MIC values of 12.5 and 50 *µ*g/ml, respectively. Bioactive compounds sapintoxin A (3.12 *µ*g/ml), sapintoxin B (12.5 *µ*g/ml), sapintoxin C (25 *µ*g/ml), 12-(2′-N-methylaminobenzoyl)-4*α*-deoxy-5,20-dihydroxyphorbol-13-acetate (25 *µ*g/ml), 12-(2-methylaminobenzoyl)-4-deoxyphorbaldehyde-13-acetae (25 *µ*g/ml), and 12-(2-N-methylaminobenzoyl)-4*β*,5,20-trideoxyphorbol-13-acetate (50 *µ*g/ml) were isolated from* Sapium indicum* L. From* Sesbania grandiflora* (L.) Poir., isovestitol (50 *μ*g/ml), medicarpin (50 *μ*g/ml), and sativan (50 *μ*g/ml) were isolated as active compounds. Tiliacorinine, 2′-nortiliacorinine, tiliacorine, and 13′-bromo-tiliacorinine from* Tiliacora triandra* (Colebr.) Diels exhibited MIC ranging between 0.7 and 6.2 *µ*g/ml. From* Uvaria valderramensis* Cabuang, Exconde & Alejandro, valderramenols A, grandiuvarone, and reticuline were isolated and showed MICs of 10, 32, and 32 *µ*g/ml, respectively. Globospiramine obtained from* Voacanga globosa* Merr. exhibited 4 and 5.2 MICs. Compound nummularines H isolated from* Ziziphus mauritiana* Lam. exhibited MIC of 4.5 *μ*g/ml. 6-Gingerol from* Zingiber officinale* Roscoe demonstrated MIC value of 33 *μ*g/ml.

Generally, some of the reviewed plant species such as* Alpinia galanga*,* Artocarpus rigidus*,* Camchaya calcarea*,* Combretum griffithii*,* Cordia globifera*,* Croton kongensis*,* Dendrolobium lanceolatum*,* Derris indica*,* Diospyros glandulosa*,* Diospyros ehretioides*,* Friesodielsia discolor*,* Garcinia mangostana*,* Goniothalamus laoticus*,* Hedychium ellipticum*,* Marsypopetalum modestum*,* Morinda citrifolia*,* Orthosiphon stamineus*,* Piper sarmentosum*,* Polyalthia cerasoides*,* Premna odorata*,* Sapium indicum*,* Tiliacora triandra*,* Trigonostemon reidiodes*,* Voacanga globosa*,* Tinospora crispa*,* Vitex trifolia*, and* Ziziphus mauritiana* proved to be potential source of anti-TB (crude and/or bioactive compound exhibited anti-TB activities at MIC values ranging from 0 to <10 *µ*g/ml) and as such should be considered for further development as either crude extract that will be consumed as complementary or alternative TB drug or as potential bioactive compounds for the development of novel anti-TB drug.

## 7. Conclusion

There has been an increase in demand for the phytopharmaceuticals worldwide due to the fact that allopathic drugs have more side effects. This review makes an attempt to compile some of the anti-TB plants of Southeast Asian origin from wide range of families and genera that have exhibited significant* in vitro* anti-TB activities and a number of bioactive compounds from different groups of chemicals have been isolated. As stated earlier, about 2 million individuals worldwide die from TB yearly. Therefore, the findings may encourage numerous researchers to embark on the project that potentially leads to the development of standardized crude extracts that will be consumed as either complementary or alternative TB drug. The findings might as well motivate various researchers to undertake the project that may further identify and characterize the active components from these plant species in order to search for the novel natural product leads useful for new anti-TB drug discovery and development.

## Figures and Tables

**Table 1:** Southeast Asian medicinal plants screened for anti-TB.

Scientific name	Family	Local name	Part used: extract/active compound	Activity	Traditional uses (references)
*Abrus precatorius* L.	Leguminosae	Akar saga	Not stated	The extract exhibited at 500 *µ*g/ml concentration of 90.0% and inhibition against both H37R*v *and MDR strain in LRP assay [47].	Bronchitis, cough, TB [18].
			Aerial part: dichloromethane/Abruquinone B (**1**) was isolated from the extract	Compound** 1 **obtained showed anti-TB activity against H37Ra strain with MIC of 12.5 *µ*g/ml [48].	

*Abutilon indicum* (L.) Sweet	Malvaceae	Giling-gilingan	Leaves: dichloromethane and methanol/*β*-amyrin 3-palmitate (**1**), squalene (**2**), *β*-sitosterol (**3**), and stigmasterol (**4**) were isolated from the extract	All the isolated compounds (**1**, **2** and a 1 : 1 mixture of **3** and **4**) showed inhibition at MIC of >128 *µ*g/ml [49].	Cough and leprosy [49].

*Acanthus ebracteatus *Vahl.	Acanthaceae	Nguag-plaa-moa	Leaves, stem: chloroform methanol water	All the chloroform, methanol, water extract of leaves and stem showed activity against H37Ra strain in MABA at MIC of 1000 *µ*g/ml [50].	Asthma, cough [51, 52].

*Aegle marmelos *(L.) Correa	Rutaceae	Mak toum	90% ethanol	The extract was active against H37R*v* at MIC 54.88 *µ*g/ml [53].	Cough, respiratory infection, intermittent fever [53–55].
			Fruits and flowers: 90% ethanol	The activity against H37R*v* strain in MABA was observed at the MIC of 47.8 to >100 *µ*g/ml from 90% ethanolic extract [56].	

Ageratum conyzoides L.	Asteraceae	Babadaton	Whole plant: 80% methanol	Methanolic extract of whole plant exhibited inhibition against H37R*v* at the MIC 1600 *µ*g/ml in TEMA [57].	Asthma, pneumonia, fever [58, 59].

*Aglaia erythrosperma* Pannell	Meliaceae		Fruits and leaves: 95%/cabraleadiol (**1**), cabraleahydroxylactone (**2**), ethyl eichlerianoate (**3**), eichlerialactone (**4**), aglinin A (**5**), cabralealactone (**6**), aglaialactone (**7**), flavagline (**8**) were isolated from the extract	Compounds **3**, **4,7 **showed anti-TB activity against H37Ra strain with the MIC of 25 *µ*g/ml which is better than compounds **1**, **2**, **6,8 **with the MIC value of 50 *µ*g/ml, while compound **5** showed weaker activity (MIC, >200 *µ*g/ml) [60].	Nil.

*Allium odorum* L.	Liliaceae	Kucai	Leaves: 80% methanol	The extract was active against H37Rv strain at MIC 1600 *µ*g/ml [57].	Asthma, cough [61].

*Aloe vera *L.	Aloaceae	Lidah buaya	Leaves: 80% methanol	The extract showed activity against H37Rv strain at the MIC of 1600 *µ*g/ml [57].	Asthma, bronchitis [62].

*Alpinia galanga* (L.) Sw.	Zingiberaceae	Khaa, Lengkuas,	Rhizome: chloroform methanol water Compound 1′acetoxychavicol acetate (**1**) was isolated from chloroform extract of rhizome	The crude chloroform, methanol, water extract of the rhizomes exhibited activity against H37Ra strain at MIC of 0.12, 1000, and 1000 *µ*g/ml, respectively. The MIC value for the isolated compound (**1**) was 0.024 *µ*g/ml [50].	TB, bronchitis, pain in chest, whooping cough, asthma, sore throat [63–66].
			Leaves: 80% methanol	The extract showed inhibition against H37R*v* strain at the MIC 1600 *µ*g/ml [57].	

*Alpinia purpurata* K. Schum.	Zingiberaceae	Luyang pula	Leave: methanol/compounds *β*-sitosteryl-*β*-D-galactoside (**1**), *β*-sitosteryl-3-O-6′-palmityl-*β*-D-glucoside (**2**), kumatakenin (**3**) were isolated from the extract	At 100 *µ*g/ml, the methanolic extract of leaves showed 90% inhibition against H_37_Rv. All isolated compounds **1**, **2, 3 **showed MIC > 128 *µ*g/ml [67].	Cough [68].

*Alpinia zerumbet* (Pers.) B. L. Burtt & R. M. Sm.	Zingiberaceae		Rhizomes: methanol	At 100 *µ*g/ml, the methanolic extract of rhizomes showed 80% inhibition against H37Rv [67].	Common cold [68].

*Alstonia scholaris *(L.) R. Brown	Apocynaceae	Dita	Leaves: methanol/19,20E-vallesamine (**1**), a mixture of angustilobine B N_4_-oxide (**2**), N_4_-methyl angustilobine B (**3**), 20S-tubotaiwine (**4**), 6,7-seco-angustilobine B (**5**), (+)-manilamine (**6**) were obtained from the extract	Only **4** exhibited activity at MIC of 100 *µ*g/ml, against, while all other compounds like **1**, **2**, **3**, **5,6 **showed activity with >128 *µ*g/ml [69].	Fever [69].

*Amaranthus tricolor* L.	Amaranthaceae	Bayam	Whole plant: 80% methanol	Methanolic extract of whole plant exhibited inhibition against H37R*v* strain at the MIC 1600 *µ*g/ml in TEMA [57].	Cough [70].

*Andrographis paniculata* Nees	Acanthaceae	Hempedu Bumi	Herbs: aqueous	At 5 mg/ml the extract exhibited 100.0% inhibition against H37R*v* and 93.7% against MDR strain [71].	Leprosy, sore throat [72].

*Angiopteris evecta* (J. R. Forst.) Hoffm.	Marattiaceae	Paku gajah	Leaves: 80% methanol	The extract exhibited activity against H37Rv strain at the MIC of 400 *µ*g/ml [57].	Cough, fever [73].

*Anisochilus harmandii* Doan ex Suddee & A. J. Paton	Lamiaceae	Chroomuay	Aerial part: hexane and EtOAc/pimaric acid (**1**), 9*α*-13*α*-epidioxyabiet-8(14)-en-18-oic acid (**2**), 15-hydrooxydehydroabietic acid (**3**) were isolated from the extract	**1**, **2**, **3** all showed anti-TB activity at MIC, 50 *µ*g/ml [74].	Nil.

*Annona muricata* L.	Annonaceae	Sirsak	Leaves: aqueous	The extract at concentration of 5 mg/ml showed 82.1% inhibition against H37R*v* and 50.0% against MDR strain [71].	Asthma, cough [75, 76].

*Annona reticulata *L.	Annonaceae	Kantaloht (peurak)	90% ethanol	The crude extract exhibited activity at the MIC of 49.17 *µ*g/ml against H37R*v* [53].	Cough, high fever [53, 77].

*Anomianthus dulcis* (Dunal) J. Sinclair	Annonaceae	Num Wua	Stem bark: 80% ethanol, dichloromethane and water/(2S)-5-hydroxy-6,7-dimethoxyflavanone (**1**), 9-methoxyliriodenine (**4**), Liriodenine (**5**) were isolated from the extract	Compounds (**5**), (**1**), (**4**) demonstrated anti-TB activity against H37Ra using MABA with the MIC values of 100, 200, 200 *µ*g/ml, respectively [78].	Fever [78].

*Artocarpus lakoocha* Roxb.	Moraceae	Ma-Haad	Roots: dichloromethane/Lakoochins A (**1**) and B (**2**) were isolated from the extract	Compound **1** and **2** showed anti-TB activity against H37Ra strain with the MICs of 12.5 and 50 *µ*g/ml, respectively [79].	Sore throat [80].

*Artocarpus rigidus subsp. Rigidus*	Moraceae	Tampang	Root bark: flavonoid 7-demethylartonol E (**1**), chromone artorigidusin (**2**), xanthone artonol B (**3**), flavonoid artonin F (**4**), flavonoid cycloartobiloxanthone (**5**), xanthone artoindonesianin C (**6**), all isolated from n-hexane, chloroform, methanol extracts	All the isolated compounds showed activity again H37Ra in MABA with compound **4** being the most active compound (MIC 6.25 *µ*g/ml). This was followed by compounds **2** and **6 **(MIC 12.5 *µ*g/ml) and compounds **5**, **1,3 **with the MIC of 25, 50, 100 *µ*g/ml, respectively [81].	Asthma, cough [82].

*Averrhoa bilimbi* L.	Oxalidaceae	Belimbing masam	Fruits, leaves: 80% methanol	The methanol extracts of both fruit and leaves exhibited inhibition against H37R*v* at the MIC 1600 *µ*g/ml in TEMA [57].	Whooping cough and fever [83].

*Barleria lupulina *Lindl.	Acanthaceae	Sa-let-pangpon	Leaves: chloroform methanol water	Chloroform, methanol, water extract of leaves all exhibited activity against H37Ra strain at the MIC of 1000 *µ*g/ml.	Cough, fever [84, 85].
			Stem: chloroform methanol water	Chloroform extract of stem exhibited inhibition at MIC of 500 *µ*g/ml against H37Ra strain while methanol and water extract showed activities at 1000 *µ*g/ml [50].	

*Blumea balsamifera* DC.	Asteraceae	Sembung utan	Not stated	At 500 *µ*g/ml, the extract exhibited 96.0 and 82.0% inhibition against H37R*v *and MDR, respectively, in LRP assay [47].	Cough [86].

*Boesenbergia pandurata* (Roxb.) Schltr.	Zingiberaceae	Krachai	Rhizome: chloroform methanol water	Both methanol and water extracts of rhizome exhibited inhibitory activity in MABA against H37Ra strain at the MIC of 62.5 *µ*g/ml, while chloroform extract showed activity at 1000 *µ*g/ml MIC [50].	Cough [87].

*Camchaya calcarea* Kitamura	Asteraceae		Whole plant: dichloromethane/goyazensolides (**1**), lychnophorolides A (**2**), centratherin or lychnophorolides B (**3**), isogoyazensolides (**4**), isocentratherin (**5**), 5-epi- isogoyazensolides (**7**), 5-epi-isocentratherin (**8**), 1(10),E,4Z,11(13)-germacratriene-12,6-olide-15-oic acid (**9**), caffeic acid methyl ester (**10**) were isolated from the extract	Compound **4** was the most active against H37Ra strain with MIC value of 1.5 *µ*g/ml, followed by compounds **1**, **2**, **5**, **7,8** with the same MIC value of 3.1 *µ*g/ml. Compounds **3**, **9,10 **showed less activities with the MIC values of 6.2, 50, and 25 *µ*g/ml, respectively [88].	Nil.

*Capsicum annum* L.	Solanaceae	Cili	Fruit: 80% methanol	The extract showed activity against H37Rv strain at the MIC of 1600 *µ*g/ml [57].	Cough, anorexia, asthma, sore throat [89].

*Casearia grewiifolia* Vent.	Flacourtiaceae	Kruai pa	Stem bark: hexane and dichloromethane/bioactive compounds, Caseargrewiin A (**1**), Caseargrewiin B (**2**), Caseargrewiin C (**3**), Caseargrewiin D (**4**), rel-(2S,5R,6R,8S,9S,10R,18S,19R)-18,19-diacetoxy-18,19-epoxy-6-methoxy-2-(2-methylbutanoyloxy)cleroda-3,13(16),14-triene (**5**) and rel-(2S,5R,6R,8S,9S,10R,18S,19R)-18,19-diacetoxy-18,19-epoxy-6-hydroxy-2-(2-methylbutanoyloxy)cleroda-3,13(16),14-triene (**6**) were isolated from the extract	**1**, **2**, **4**, **5,6** all exhibited good anti-TB activity against H37Ra strain with MICs of 12.5 *µ*g/ml while **3** showed moderate activity with MIC of 25 *µ*g/ml [90].	Fever [90].

*Catharanthus roseus* (L.) G. Don	Apocynaceae	Kemunting cina	Leaves: 80% methanol	The extract was active against H37R*v* at MIC 1600 *µ*g/ml [57].	Asthma, TB [91].

*Ceiba pentandra* (L.) Gaertn.	Bombacaceae	Kabu	Fruit: 80% methanol	The extract exhibited anti-TB activity against H37Rv strain at the MIC of 1600 *µ*g/ml [57].	Bronchitis, fever [92].

*Centella asiatica* (L.) Urb.	Apiaceae	Pegaga	Whole plant: 80% methanol	The extract showed activity against H37Rv strain at the MIC of 1600 *µ*g/ml [57].	TB, leprosy, asthma [93].
			Herbs: aqueous	78.5 and 50.0% inhibition were observed at 5 mg/ml concentration against H37Rv and MDR, respectively [71].	

*Chisocheton penduliflorus* Planch. ex Hiern	Meliaceae		Wood and leaves: 95% methanol/cabraleadiol (**1**), allo-aromadendrane-10*β*, 14-diol (**2**), allo-aromadendrane-10*α*, 14-diol (**3**) eichlerialactone (**4**), cabraleahydroxylactone (**5**), cabralealactone (**6**), allo-aromadendrane-10*β*,13,14-triol (**7**) were isolated from the extract	Compound** 4 **showed good anti-TB activity against H37Ra with the MIC value of 25 *µ*g/ml, better than **1**, **2**, **5**, **6,7** with the MICs of 50 *µ*g/ml, while compound **3** showed weaker activity (MIC, 100 *µ*g/ml) [94].	Nil.

*Chromolaena odorata* (L.) R. M. King & H. Rob.	Asteraceae	Agonoi	Flowers: isosakuranetin (**1**), 4′-hydroxy-5,6,7-trimethoxyflavanone (**2**), acacetin (**3**), luteolin (**4**), all isolated from chloroform extract	The compounds isolated exhibited activities against H37Ra at different MIC values (*µ*g/ml) 174.8 (**1**), 606.0 (**2**), 704.2 (**3**), 699.3 (**4**) [95].	Cough [96].

*Citrus aurantiifolia* (Christm.) Swingle	Rutaceae	Limau nipis	Not stated	In LRP assay, extract at 500 *µ*g/ml showed 98.0% inhibition against H37R*v* strain and 36.0% against MDR strain [47].	Sore throats, bronchitis, asthma [97, 98].

*Citrus microcarpa* Bunge	Rutaceae	Limau kasturi	Leaves: 80% methanol	The extract exhibited anti-TB activity against H37Rv strain at the MIC of 1600 *µ*g/ml in TEMA [57].	Cough [99].

*Clausena excavate* Burm. f.	Rutaceae	Sun Soak	Dentatin (**1**), nordentatin (**2**), clausenidin (**3**), O-methylated clausenidin (**4**), 3-formylcarbazole (**5**), mukonal (**6**), 3-methoxycarbonylcarbazole (**7**), 2-hydroxy-formyl-7-methoxycarbazole (**8**), clauszoline (**9**); compounds **1**, **2**, **6**, **7**, **8,9** where isolated from the chloroform extract of rhizomes; compound **3 **was isolated from crude hexane extract of rhizome. Methylation of compound **3** gave rise to compound **4**; compound **10** was isolated from crude chloroform extract of the root	Compounds **1**, **4,7,** isolated, were more active against H37Ra with MIC of 50 *µ*g/ml; this is followed by **2**, **5**, **8,9** with MIC of 100 *µ*g/ml, while compounds **3** and **6** are with MIC of 200 *µ*g/ml [100].	TB [101].

*Clausena guillauminii* Tanaka	Rutaceae		Roots: acetone/fluroclausine A (**1**) and heptazoline (**2**) were obtained from the extract	Using the green fluorescent protein microplate assay (GFPMA), both** 1** and **2** exhibited anti-TB activity against H37Ra strain with the same IC_50_ value of 25 *µ*g/ml [102].	Cough [103].

*Clausena harmandiana *(Pierre) Guillaumin	Rutaceae	Song Fa	Fruits and flowers: 90% ethanol	MICs of 83.1 to >100 *µ*g/ml were observed when 90% ethanolic extract of fruits and flowers was tested against H37R*v* in MABA [56].	Cough [103].

*Clerodendrum indicum* (L.) Kuntze	Verbenaceae	Bunga pagoda	Flowers: 80% methanol	The extract exhibited anti-TB activity against H37Rv strain at the MIC of 1600 *µ*g/ml [57].	Cough, asthma [104].

*Clitoria ternatea* L.	Leguminosae	Bunga kelentik	Whole plant: 80% methanol	At MIC = 1600 *µ*g/ml, the extract showed anti-TB activity against H37Rv strain [57].	Asthma, leprosy, TB [105].

*Coccinia grandis* (L.) Voigt	Cucurbitaceae	Phak tamlueng	Leaves: chloroform methanol water	In MABA, chloroform, methanol, water extract of leaves all exhibited activity against H37Ra strain at the MIC of 1000 *µ*g/ml [50].	Asthma, cough, bronchitis [106, 107].

*Coleus atropurpureus *L. Benth	Lamiaceae	Piladang	Compound 2′,5′-dimethyl benzopelargonolactone was isolated from the chloroform fraction of leaf	The isolate was active against H37R*v* strain at the MIC of 200 *µ*g/ml [108].	Bronchitis, TB [108].

*Colocasia esculenta* (L.) Schott	Colocasieae	Keladi cina	Leaf: 80% methanol	The methanol extract of leaves showed activity against H37R*v* strain with the MIC value 1600 *µ*g/ml [57].	Asthma, coughing with sputum [109].

*Combretum griffithii* Van Heurck & Müll. Arg.	Combretaceae	Khamin khruea	Stem: methanol/1-(2-hydroxy-4-methoxyphenyl)-3-(4-hydroxy-3-methoxyphenyl)propane (**1**) were isolated from the extract	**1** exhibited anti-TB activity against H37Ra strain with the MIC of 3.13 *µ*g/mL [110].	Coughing, leprosy [111].

*Cordia globifera* W. W. Smith	Boraginaceae	Sak Hin	Root: dichloromethane/Globiferin (**1**), cordiachrome B (**2**), cordiachrome C (**3**), cordiaquinol C (**4**), alliodorin (**5**), elaeagin (**6**), cordiachromene (**7**) were isolated from the extract	Compounds **1**, **2,3** displayed good activity (MIC, 6.2, 12.5, 1.5 *µ*g/ml resp.) using MABA against H_37_Ra, followed by **5**, **6,7** (MIC, 12.5 *µ*g/ml) and then **4** with MIC of 25 *µ*g/ml [112].	Cough [113].

*Costus speciosus* (J. Koenig) Sm.	Costaceae	Setawar halia	Stem and flowers: 80% methanol	The methanol extract of Stem and flowers exhibited anti-TB activity against H37R*v* strain with the MIC value 800 *µ*g/ml [57].	Asthma, bronchitis [114].

*Croton kongensis* Gagnep.	Euphorbiaceae	Plao Ngeon, Kh*ổ* sâm bắc bộ	Leaves: crude dichloromethane extract. *ent*-8,9-*seco*-7*α*,11*β*-diacetoxykaura-8(14),16-dien-9,15-dione (**1**), *ent*-8,9-*seco*-8,14-epoxy-7*α*-hydroxy-11*β*-acetoxy-16-kauren-9,15-dione (**2**), *ent*-8,9-*seco*-7*α*-hydroxy-11*β*-acetoxykaura-8(14),16-dien-9,15-dione (**3**), all were isolated from dichloromethane extract of leaves	Dichloromethane crude extract exhibited activity against H37Ra strain with the MIC value 12.5 *µ*g/ml in MABA. The isolated compounds **2** and **3** showed better activity with the MIC value 6.25 *µ*g/ml than compound **1** with 25.0 *µ*g/ml MIC [115].	Leprosy, weight loss [116].
			Whole plants, leaves: ethanol, ethyl acetate, methylene chloride, n-Hexane/ent-1*β*,7*α*,14*β*-triacetoxykaur-16-en-15-one (**1**), ent-7*α*,18-dihydroxykaur-16-en-15-one (**2**), ent-16(S)-18-acetoxy-7*α*- hydroxykaur-15-one (**3**), ent-18-acetoxy-7*α*-hydroxykaur-16-en-15-one (**4**), ent-1*β*,14*β*-diacetoxy-7*α*-hydroxykaur-16-en-15-one (**5**), ent-1*β*-acetoxy-7*α*,14*β*-dihydroxykaur-16-en-15-one (**6**), ent-7*α*,14*β*-dihydroxykaur-16-en-15-one (**7**) were all isolated from ethanol, ethyl acetate, methylene extract of whole plants and leaves	The activities against H37Ra strain were observed at the MIC of 25–50, >50, 6.25–12.5, 12.5–25 *µ*g/ml from ethanol, ethyl acetate, methylene chloride, n-Hexane crude extracts of whole plants and leave using microtiter resazurin assay. Isolated compound **1** exhibited the highest activity with MIC values of 0.78, 1.56, 3.12–12.5 *µ*g/ml against H37Ra, H37Rv, other resistant strains of *M. tb* screened. Both compounds **2 **and** 3 **exhibited MIC at 1.56 *µ*g/ml. Compounds **4**, **5**, **6,7** on the other hand showed MIC value 3.12–6.25 against H37Ra and H37Rv and other resistant strains of *M. tb* [117].	

*Curcuma aeruginosa* Roxb.	Zingiberaceae	Waan mahaamek	Rhizomes: water/essential oil	Using GFPMA, essential oil showed weaker anti-TB activity against H37Ra strain with 2500 *µ*g/ml MIC value [118].	Dyspepsia [118].

*Dalbergia parviflora* Roxb.	Leguminosae	Sak Kee	Stem: hexane, ethyl acetate, methanol/Flavanone (**1**), dalparvone (**2**), dalparvinene (**6**) were isolated from the extract	Using GFPMA, compound **1** exhibited good anti-TB activity against H37Ra strain with the MIC of 12.5 *µ*g/ml, while compounds **2** and **6** showed activity with the MICs of 50 *µ*g/ml [119].	Expectorant [119].

*Dasymaschalon dasymaschalum* (Blume) I. M. Turner	Annonaceae	Buu ngong	Leaves: ethyl acetate/3*β*-hydroxy-21-O-acetyl-24-methylenecycloartane (**1**) was obtained from the extract	Compound **1** displayed activity against H37Ra strain with the MIC value of 50 *µ*g/ml [120].	Nil.

*Dendrolobium lanceolatum* (Dunn) Schindl.	Leguminosae	Kraduk-Khiat	Root: hexane and dichloromethane/Flavanones 1 (**1**), flavanones (**2**), flavan (**3**), dibenzocycloheptene derivative (**4**), 4′-hydroxy-7,8-(2′′,2′′-dimethylpyran)flavan (**5**) isolated from the extract.	Compound **1** exhibited highest activity against H37Ra strain with MIC of 6.3 *µ*g/ml, followed by **2** (MIC, 12.5 *µ*g/ml) and then **3** and **5** with MICs of 25 *µ*g/ml [121].	Nil.

*Derris indica* L	Leguminosae		Root and stem: dichloromethane extract/3-methoxy-(3′′,4′′-dihydro-3′′,4′′-diacetoxy)-2′′,2′′-dimethylpyrano-(7,8:5′′,6′′)-flavone (**1**), 2′-methoxy-4′,5′-methylenedioxyfurano [7,8:4′′,5′′]-flavone (**2**), 8,4′-dimethoxy-7-O-*γ*,*γ*-dimethylally-lisoflavone (**3**), 3,4-methylenedioxy-10-methoxy-7-oxo[2]benzopyrano[4,3-b]benzopyran (**4**), desmethoxy kanugin (**5**), karanjin (**6**), lacheolatin B (**7**), pongachromene (**8**), 3,7-dimethoxyflavone (**9**), pachycarin D (**10**), maackiain (**11**), medicarpin (**12**) karanjachromene (**13**), pinnatin (**14**) isolated from this extract	**4**, **13,14** (6.25, 12.5, 12.5 *µ*g/ml, resp.) showed stronger activity compared to **1**, **5**,** 7**, **9**, **10** (25, 50, 50, 50, 50 *µ*g/ml resp.) that showed moderate activity, with **3**, **8**, **10,12** (100, 100, 200, 100 *µ*g/ml resp.) showing weaker activity against H37Ra strain using MABA [122].	Bronchitis and whooping cough [23].

*Diospyros decandra* Lour.	Ebenaceae	Chan	Betulinic acid (**1**) and 2-oxo-3*β*,19*α*-dihydroxy-24-nor-urs-12-en-28-oic acid (**2**)	**1** and **2 **exhibited moderate to weak anti-TB activity against H37Ra strain with MIC of 25 and 200 *µ*g/ml, respectively [123].	Fever [123].

*Diospyros ehretioides* Wall. ex G. Don	Ebenaceae	Nom ngua	Fruits: dichloromethane/palmarumycins JC1 (**1**), palmarumycins JC2 (**2**), isodiospyrin (**3**), isodiospyrol A (**4**) were isolated from the extract	Compound** 2 **exhibited good (MIC = 6.25 *µ*g/ml) anti-TB activity against H37Ra, followed by compound **4 **(MIC = 50 *µ*g/ml). Compounds **1 **and **3** showed weak activity (MICs ≥ 200 *µ*g/ml) [124].	Nil.

*Diospyros glandulosa* Lace	Ebenaceae	Kluai ruesi	Diospyrin, isolated from dichloromethane extract of wood	Diospyrin isolated showed activity against H37Ra in MABA with the MIC of 6.25 *µ*g/ml [125].	Nil.

*Diospyros rhodocalyx* Kurz	Ebenaceae	Tako Na	Betulinaldehyde, obtained from dichloromethane extract of wood	Betulinaldehyde exhibited inhibitory activity in microbroth dilution assay against H37Ra with the MIC of 25 *µ*g/ml [125].	Nil.

*Eclipta prostrata *(L.) L.	Asteraceae	Kra-meng	Whole plant: chloroform methanol water	Water, chloroform, methanol extracts of whole plant exhibited inhibition in MABA against H37Ra strain at the MICs of 62.5, 125, 1000 *µ*g/ml respectively [50].	Asthma and TB [126].

*Eriosema chinense* Vogel	Leguminosae	Toon Khonklong	Roots: hexane, dichloromethane, methanol/Khonklonginol A (**1**), B (**2**), F (**6**), H (**8**), lupinifolinol (**9**), dehydrolupinifolinol (**10**), flemichin D (**11**), eriosemaone A (**12**), lupinifolin (**13**) were obtained from the extract	Crude hexane extract showed anti-TB activity against H37Ra strain with the MIC value of 50 *µ*g/ml using MABA. Compounds **10**, **11**, **12,13 **demonstrated good anti-TB activity against H37Ra strain with the same MIC value of 12.5 *µ*g/ml. Compounds **1**, **8,9** exhibited moderate activity with the same MIC value of 25 *µ*g/ml. Compounds **2** and **6** showed activity at MIC values of 50 and 100 *µ*g/ml, respectively [127].	Nil.

*Erythrina fusca* Lour.	Fabaceae	Thong long	Stem bark: hexane and ethyl acetate/sandwicensin (**1**), erythrisenegalone (**2**), lonchocarpol A (**3**), lupinifolin (**4**) were isolated from the extract	Compounds **1**, **2**, **3,4** demonstrated anti-TB activity against H37Ra with MICs of 100, 50, 50, 25 *µ*g/ml, respectively [128].	Antibacterial [129].

*Erythrina subumbrans* Merr.	Leguminosae		Bark: n-hexane, dichloromethane and methanol/1-methoxyerythrabyssin II (**1**) was isolated from the extract Stem: n-hexane and dichloromethane/erystagallin A (**1**), erycristagallin (**2**), 5-hydroxysophoranone (**3**), erysubin F (**4**) were isolated from the extract	Compound **1** showed activity against H37Ra strain with 50 *µ*g/ml MIC value [130]. All compounds **1**, **2**, **3,** 4 exhibited anti-TB activity against H37Ra with the MICs of 12.5 *µ*g/ml [131].	Cough [131].

*Etlingera elatior* (Jack) R. M. Sm.	Zingiberaceae	Bunga kantan	Rhizomes: methanol/Stigmasterol (**1**) and *β*-sitosterol (**2**) were isolated from the extract	At 100 *µ*g/ml, the methanolic extract of rhizomes showed 86% inhibition against H37Rv. The isolated compounds **1** and **2** exhibited MIC > 128 *µ*g/ml [67].	Nil.

*Etlingera pavieana* (Pierre ex Gagnep.) R. M. Sm.	Zingiberaceae		Rhizomes: dichloromethane/(E)-((E)-3-(4-methoxyphenyl)allyl)3-(4-hydroxyphenyl)acrylate (**1**) was isolated from the crude extract	Compound **1** demonstrated anti-TB activity with the MIC value of 50.00 *µ*g/ml [132].	Fever and cough [132].

*Fernandoa adenophylla *(Wall. Ex G. Don) Steenis	(Bignoniaceae)	Khae Pa	Fruits and flowers: 90% ethanol	In microbroth dilution assay, the extract exhibited inhibition at MIC of 79.7 to >100 *µ*g/ml [56].	Skin diseases [133].

*Feroniella lucida *Swingle	Rutaceae	Sung (mak)/kohk sung	90% ethanol	The extract showed inhibition against H37R*v* at MIC value 91.54 *µ*g/ml in MABA [53].	Cough, TB [53, 134].
			Fruits and flowers: 90% ethanol	In MABA, 90% ethanolic extract of fruits and flowers exhibited activity against H37Rv strain with MIC ranging from 90.4 to >100 *µ*g/ml [53].	

*Ficus carica* L.	Moraceae	Ara	Leaves: 80% methanol	There was anti-TB activity against H37Rv strain at 1600 *µ*g/ml [57].	Asthma, cough [135].

*Flemingia strobilifera* (L.) W. T. Aiton	Fabaceae	Serengan	Leaves: 80% methanol	At MIC = 1600 *µ*g/ml, the extract showed anti-TB activity against H37Rv strain [57].	TB [136].

*Friesodielsia discolor* (Craib) D. Das	Annonaceae		Leave: dichloromethane and ethyl acetate/3′-formyl-2′,4′-dihydroxy-6′-methoxychalcone (**1**) was isolated from the extract	Compound **1** exhibited anti-TB activity against H37Ra strain with 6.25 *µ*g/ml MIC value [137].	Nil.

*Garcinia mangostana *L.	Clusiacea	Mangkhud	Fruits: *α*-mangostin (**1**), *β*-mangostin (**2**), *γ*-mangostin (**3**), garcinone D (**4**), mangostenol (**5**), garcinone B (**6**), mangostanin (**7**), mangostanol (**8**), mangostenone A (**9**), tovophyllin B (**10**), demethylcalabaxanthone (**11**), trapezifolixanthone (**12**), mangostinone (**13**) all isolated from chloroform, methanol extract	Compounds isolated showed activities against H37Ra in microbroth dilution at different MIC values (*µ*g/ml) 6.25 (**1**), 6.25 (**2**), 25 (**3**), 25 (**4**), 100 (**5**), 6.25 (**6**), 25 (**7**), 200 (**8**), 25 (**9**) 25 (**10**), 12.5 (**11**), 12.5 (**12**), 200 (**13**) [138].	Fever [139].

*Glycos*mis *pentaphylla* (Retz.) DC.	Rutaceae	Xom Xeuan	Fruits and flowers: 90% ethanol	The 90% ethanol extracts of fruits and flowers exhibited activities against H37R*v* strain with the MIC of 93.5 to >100 *µ*g/ml in MABA [56].	Cough [140].

*Goniothalamus gitingensis* Elmer	Annonaceae		Leaves/Liriodenine (**1**) was isolated from the extract	**1** showed anti-TB with the MIC of 16 *µ*g/ml [141].	Nil.

*Goniothalamus laoticus* (Finet & Gagnep.) Bân	Annonaceae	Khao-Lam-dong	Flowers: goniotriol (**1**), (+)-altholactone (**2**), howiininA (**3**) and an aporphine alkaloid; (−)-nordicentrine (**4**), all isolated from n-hexane, ethyl acetate, methanol extracts	In microbroth dilution, the isolated compound **4** showed the best activity against H37Ra with MIC of 12.5 *µ*g/ml, followed by compounds **2** and **3** both with the MIC of 6.25 *µ*g/ml, and then compound **1** with 100 *µ*g/ml [142].	Cold [143].

*Gynura divaricata* (L.) DC.	Asteraceae		Leaves: hexane, dichloromethane, methanol/essential oil	The essential oil showed inhibitory effect against H37Ra strain with the MIC value of 50 *µ*g/ml [144].	Bronchitis and pulmonary TB [144].

*Gynura pseudochina* (L.) D.C. var. hispida Thv.	Asteraceae	Waan Mahaakaan	Whole plant: chloroform methanol water	At the MIC of 200 *µ*g/ml, chloroform extract of whole plant exhibited activity against H37Ra strain while both methanol and water extracts exhibited activity at 1000 *µ*g/ml MIC, respectively [50].	Asthma, fever, AIDS [145, 146].

*Haplophragma adenophyllum (Wall. ex G. Don) Dop*	Bignoniaceae	Kay pa	90% ethanol	In MABA, the MIC of 83.25 *µ*g/ml was observed against H37R*v* [53].	Cough [53].

*Hedychium ellipticum* Buch.-Ham. ex Sm.	Zingiberaceae		Rhizomes: n-hexane and dichloromethane/Coronarin E (**1**) and 16-Hydroxylabda-8(17),11,13-trien-15,16-olide (**7**) were isolated from the extract	Compounds** 1 **and** 7 **exhibited good anti-TB activity against H37Ra strain using GFPMA with the MIC value of 12.5 and 6. 25 *µ*g/ml, respectively [147].	Bronchitis [148].

*Heliotropium indicum* Linn.	Boraginaceae	Yaa Nguang Chaang	Leaves: water	The crude extract showed activity against H37Ra strain using MABA with the MIC of 20.8 *µ*g/ml [149].	Asthma [149].

*Hibiscus rosa-sinensis* L.	Malvaceae	Bunga raya	Leaves: 80% methanol	The extract exhibited anti-TB activity against H37Rv strain at the MIC of 1600 *µ*g/ml [57].	Cough, leprosy, bronchial catarrh [150].

*Hymenocardia wallichii* Tul	Euphorbiaceae		Stem: dichloromethane, methanol, hexane/Squalene were isolated from the extract	Squalene displayed anti-TB activity against H37Ra strain with the MIC value of 100 *µ*g/ml [151].	Nil.

*Hyptis suaveolens* (L.) Poit.	Lamiaceae	Maeng luk kha	Whole plant: hexane, chloroform and methanol/8*α*,9*α*-epoxysuaveolic acid (**2**), suaveolic acid (**4**), suaveolol (**5**) were isolated from the extract	Compounds **2**, **4**, **5** displayed weak anti-TB activities (MIC 100–200 *µ*g/ml) against H37Ra strain using MABA [152].	Fever and respiratory tract infections [152].

*Jasminum sambac* (L.) Aiton	Oleaceae	Melor	Leaves: 80% methanol	At MIC = 1600 *µ*g/ml, the crude extract showed inhibition against H37Rv strain [57].	Cough, Leprosy [153].

*Jatropha curcas* L.	Euphorbiaceae	Jarak	Leaves: 80% methanol	The extract showed *in vitro* activity against H37Rv strain at the MIC of 1600 *µ*g/ml [57].	Leprosy [154].

*Jatropha integerrima* Jacq.	Euphorbiaceae		Roots: dichloromethane and ethanol/caniojane were isolated from the extract	Caniojane exhibited anti-TB activity against H37Ra strain using MABA with 25 *µ*g/ml MIC value [155].	Styptic [156].

*Justicia gendarussa* Burm. f.	Acanthaceae	Urat sugi	Leaves: 80% methanol	The extract exhibited anti-TB activity against H37Rv strain at the MIC of 1600 *µ*g/ml [57].	Respiratory disorders [157].

*Kaempferia galangal* L.	Zingiberaceae	Kencur	Not stated	In LRP assay, the extract at 500 *µ*g/ml exhibited 69.0% inhibition against both H37R*v* and MDR strain [47].	Asthma, cough [158].

*Kaempferia marginata* Carey	Zingiberaceae	Tup mup	Whole plant: dichloromethane and/(1R,2S,5S,9S,10S,11R,13R)-1,2,11-trihydroxy-pimara-8(14),15-diene (**2**), (1S,5S,7R,9R,10S,11R,13R)-1,7,-11-trihydroxypimara-8(14),15-diene (**3**), (1R,2S,5S,7S,9R,10S,13R)-1,2-dihydroxypimara-8(14),15- diene-7-one (**6**), sandaracopimaradien-1*α*-ol,2*α*-acetoxysandaracopimaradien-1*α*-ol, sandaracopimaradien-1*α*,2*α*-diol were isolated from the extract	Compounds sandaracopimaradien-1*α*-ol and 2*α*-acetoxysandaracopimaradien-1*α*-ol exhibited ant-TB activity against H37Ra strain with the MIC values of 25 and 50 *µ*g/ml, respectively. Compounds **2**, **3**, **6**, sandaracopimaradien-1*α*,2*α*-diol were less active (MICs of > 100 *µ*g/ml) [159].	Fever [159].

*Lantana camara *L.	Verbenaceae	Kembang telek	Not stated	At 500 *µ*g/ml, the extract exhibited 94.0 and 79.0% inhibition against H37R*v* and MDR, respectively, in LRP assay [47].	TB, leprosy, asthma [160, 161].

*Lepisanthes rubiginosa* (Roxb.) Leenh.	Sapindaceae	Mertajam	Leaves: 80% methanol	In broth microdilution assay, the extract showed inhibition against H37Rv strain at 1600 *µ*g/ml [57].	TB [162].

*Licuala spinosa* Thunb.	Arecaceae	Palas tikus	Leaves: 80% methanol	In TEMA, the extract showed inhibition against H37Rv strain at 1600 *µ*g/ml [57].	TB [57].

*Limnophila geoffrayi* Bonati	Scrophulariaceae	Prod Ka yaeng	Flavones nevadensin (**1**) and isothymusin (**2**), all were isolated from chloroform extract of aerial part	Both compounds **1** and **2** showed activities against H37Ra in MABA at the MIC = 200 *µ*g/ml [163].	Expectorant [164].

*Marsypopetalum modestum (Pierre) B. Xue*	Annonaceae	Tin Tang Tia	Fruits and flowers: 90% ethanol	The extract of fruits and flowers exhibited activities at MIC ranging between 0.05 and 11.9 *µ*g/ml in MABA against H37R*v* [56].	Nil.

*Micromelum minutum Wight & Arn.*	Rutaceae	Sa Mat Khao	Fruits and flowers: 90% ethanol	The activity against H37R*v* strain in MABA was observed at the MIC of 45.7 to >100 *µ*g/ml from 90% ethanolic extract [56].	Cough, fever [165].

*Momordica charantia *L.	Cucurbitaceae	Ampalaya	Leaves: ethanol/2,4-bis(2-phenylpropan-2-yl)phenol (**1**) isolated from the extract	Compound **1** exhibited anti-TB activity against H37Rv strain MIC value of 14 *µ*g/ml using MABA [166].	Leprosy [167].

*Morinda citrifolia* Linn.	Rubiaceae	Noni	Leaves: ethanol and hexane/(E)-phytol (**1**), cycloartenol (**2**), stigmasta-4-en-3-one (**3**), stigmasta-4-22-dien-3-one (**4**), *β*-sitosterol (**5**), stigmasterol (**6**), campesta-6,22-dien-5*α*,8*α*-epidioxy-3*β*-ol (**7**) were isolated from the extract	At 100 *µ*g/ml, the crude extract of ethanol and hexane fractions displayed 89 and 95% inhibition, respectively, against H37Rv strain. 2 : 1 mixture of compounds **3** and **4** exhibited good activity (MIC = 2 *µ*g/ml) against H37Rv strain followed by **7 **(MIC = 2.5 *µ*g/ml) and then **1** and **6** (MICs = 32 *µ*g/ml). Compound **2** and **5** were less active with MIC values of 64 and 128 *µ*g/ml, respectively [168].	Respiratory infection [168].

*Morus alba* L.	Moraceae	Merbatu	Leaves and fruit: 80% methanol	In broth microdilution assay, the extract showed inhibition against H37Rv strain at 1600 *µ*g/ml [57].	Cough [169].

*Murraya paniculata* (L.) Jack	Rutaceae	Kaeo	Leaves: chloroform methanol water	Chloroform extract of leaves at the MIC of 250 *µ*g/ml showed activity against H37Ra strain while both methanol and water extracts exhibited activity at 1000 *µ*g/ml MIC, respectively [50].	Cough, asthma, expectorant [170–172].

*Orthosiphon stamineus* Benth.	Lamiaceae	Misai Kuching	Leaves: hexane, chloroform, ethyl acetate	The hexane, chloroform, ethyl acetate extracts of leaves exhibited activity with the MICs of 25.00, 3.12, 6.25 *µ*g/ml [173].	Loss of weigh [174].

*Passiflora foetida* L.	Passifloraceae	Letup-letup	Whole plant: 80% methanol	There was inhibition against H37R*v* at MIC = 1600 *µ*g/ml [57].	Cough [175].

*Pedilanthus tithymaloides * (L.) Poit.	Euphorbiaceae	Sa yaek	Leaves: methanol, water, hexane/1*α*,13*β*,14*α*-trihydroxy-3*β*,7*β*-dibenzoyloxy-9*β*,15*β*-diacetoxyjatropha-5,11E-diene (**1**), 1*α*,7*β*,13*β*,14*α*-tetrahydroxy-3*β*-benzoyloxy-9*β*,15*β*-diacetoxyjatropha-5,11E-diene (**2**), 1*α*,8*β*,9*β*,14*α*,15*β*-pentaacetoxy-3*β*-benzoyloxy-7-oxojatropha-5,12-diene (**3**), 7,8*β*,9*β*,14*α*,15*β*-pentaacetoxy-3*β*-benzoyloxy-1*α*,5*β*-dihydroxyjatropha-6(7),12-diene (**4**), 1*α*,7,8*β*,9*β*,14*α*,15*β*-hexaacetoxy-3*β*-benzoyloxy-5*β*-hydroxyjatropha-6(7),12-diene (**5**) were obtained from the extract	Compound **1** showed stronger (12.5 *µ*g/ml) activity compared to **2**, **3**, **4,** and **5** (100, 50, 100, and 50 *µ*g/ml resp.) against H37Ra in MABA [176].	Nil.

*Petiveria alliacea* L.	Phytolaccaceae	Singawalang	96% ethanol: leaves	The ethanolic extract of leaves exhibited activity against drug sensitive and resistant strains of H37R*v* at the MIC of 1280 *µ*g/ml [177].	Antibacterial [178].
			Not stated	At 500 *µ*g/ml, the extract exhibited 98.0 and 76.0% inhibition against H37R*v* and MDR, respectively, in LRP assay [47].	

*Phyllanthus acidus* (L.) Skeels	Euphorbiaceae	Cermai	Leaves: 80% methanol	The extract exhibited anti-TB activity against H37Rv strain at the MIC = 1600 *µ*g/ml [57].	Cough [179].

*Piper betle* L.	Piperaceae	Plu, Daun sirih	Leaves:chloroform methanol water	Chloroform extract of leaves was more active against H37Ra strain with MIC value 62.5 *µ*g/ml than the methanol and water extract both with activity at MIC of 1000 *µ*g/ml [50].	Asthma, leprosy cough, dyspnea, bronchitis [180–182].

*Piper chaba *Hunter	Piperaceae	Dee plee	Fruit: chloroform, methanol, water; compound Piperine (**1**) was isolated from chloroform crude extract of fruit	Inhibition against H37Ra strain was observed at MIC (*µ*g/ml) of 16, 25, 1000 with respect to the chloroform, methanol, water extract of fruit in MABA. Compound **1** isolated exhibited activity at MIC of 50 *µ*g/ml [50].	Asthma [183].
			Stem: n-hexane/chabamide (**1**) was isolated from the extract	Compound** 1** exhibited anti-TB activity with the MIC value of 12.5 *µ*g/ml against H37Ra strain [184].	

*Piper nigrum* L.	Piperaceae	Lada hitam	Fruit: 80% methanol	The extract exhibited anti-TB activity against H37Rv strain at the MIC of 1600 *µ*g/ml [57].	Asthma, bronchitis, TB, sore throat [185, 186].
			Leaves: ethyl acetate n-hexane water	Ethyl acetate, n-hexane, water extract fraction of leaves exhibited inhibition against H37Rv strain using TEMA at the MIC values of 25, 50, 100 *µ*g/ml, respectively [185].	

*Piper sarmentosum *Roxb.	Piperaceae	Kadok, Cha-plu	Whole plant: 80% methanol	At the MIC = 800 *µ*g/ml, the extract showed inhibition against H37Rv strain [57].	Cough [187].
			Leaves: petroleum ether, Chloroform, methanol	The methanol extract of leaves showed more activity against MTB (MIC 12.5 *µ*g/ml) in TEMA than both petroleum ether and Chloroform extracts (MIC 25 *µ*g/ml). The ethyl acetate and chloroform fractions of methanol extract exhibited MICs at 3.12 *µ*g/ml [188].	
			Leaves: aqueous, ethanol	The aqueous and ethanol extracts of leaves exhibited anti-TB activity MIC/MBC 12.5 *µ*g/ml. Methanolic extract was fractionated with ethyl acetate; the fraction of ethyl acetate exhibited anti-TB activity with MIC/MBC 3.12 *µ*g/ml [189].	
			Fruits: hexane and methanol/pellitorine (**1**), guineensine (**2**), brachystamide B (**3**), sarmentine (**4**), brachyamide B (**5**), sarmentosine (**8**), 1-(3,4-methylenedioxyphenyl)-1E-tetradecene (**11**) were isolated from the extract	Compounds **1** and **11** were more active (MICs = 25 *µ*g/ml) against H37Ra strain followed by **2**, **4, 5** (MICs = 50 *µ*g/ml); then **3** and **8** showed weaker activities with the MIC values of 100 and 200 *µ*g/ml, respectively [190].	

*Pistia stratiotes* L.	Araceae	Water lettuce	Whole plant: 80% methanol	The inhibition against H37Rv was observed at the MIC of 1600 *µ*g/ml [57].	Leprosy, TB [191].

*Pluchea indica* (L.) Less.	Asteraceae	Beluntas	Flower Leaves: 80% methanol	In TEMA, the extracts of flowers and leaves showed inhibitory activities against H37Rv strain at the MIC of 800 *µ*g/ml each [57].	TB [192].
			Leaves: aqueous	The extract showed 100% inhibition against H37Rv and MDR at 5 mg/ml [71].	

*Polyalthia cerasoides *(Roxb.) Benth. ex Bedd	Annonaceae	Sai den	Root: hexane, EtOAc, MeOH/Bidebiline E (**1**), octadeca-9,11,13-triynoic acid (**2**), *α*-humulene (**3**) were isolated from the extract	Isolated compounds **1**, **2,3** showed anti-TB activity against H37Ra strain with MICs of 6.25 *µ*g/ml [193].	Fever [193].

*Polyalthia debilis* (Piere) Finet & ganep	Annonaceae	Kon Krok	Root: methanol/Debilisone B (**1**), Debilisone C (**2**), Debilisone E (**3**)	Compounds **1**, **2**, **3** showed moderate anti-TB activity against H37Ra strain with MIC values of 25.0, 12.5, 25.0 *µ*g/ml, respectively [194].	TB [195].

*Premna odorata *Blanco	Lamiaceae	Alagaw	Leaves: methanol and dichloromethane/1-heneicosyl formate (**1**) was isolated from the extract	Crude methanolic extract showed inhibition against H37Rv strain with >128 *µ*g/ml MIC. Compound **1** showed good anti-TB activity (8 *µ*g/ml) [196].	TB [196].

*Rhoeo spathacea* (Sw.) Stearn	Commelinaceae	Nil	Leaves: 80% methanol	At 5 mg/ml, the extract exhibited 100% inhibition against H37R*v* and MDR [71].	TB, asthma [197].

*Rollinia mucosa *(Jacq.) Baill.	Annonaceae	Khanthaloht	Fruits and flowers: 90% ethanol	Fruits and flowers extract were active at MIC ranging between 43.9 and 75.2 *µ*g/ml in MABA [56].	Nil.

*Rothmannia wittii* (Craib) Bremek.	Rubiaceae	Muk Mo	Bark and fruits: n-Hexane and ethyl acetate/A compound 6*β*-hydroxy-10-O-acetylgenipin (**1**) was isolated from the extract	Compound **1** displayed activity against H_37_Ra using MABA with MIC value of 12.50 *µ*g/ml [198].	Fever [198].

*Sapium indicum* L.	Euphorbiaceae		Fruit: hexane/compounds 12-(2-*N*-methylaminobenzoyl)-4*β*,5,20-trideoxyphorbol-13-acetate (**1**), 12-(2-*N*-methylaminoben-zoyl)-4*α*,5,20-trideoxyphorbol-13-acetate (**2**), sapintoxin A (**3**), *α*- saponine (**4**), sapintoxin C (**5**), 12-(2-*N*-methylaminobenzoyl)-4*α*,20-dideoxy-5-hydroxyphorbol-13-acetate (**6**) sapintoxin B (**7**), 12-(2′-*N*-methylaminobenzoyl)-4*α*-deoxy-5,20-dihydroxyphorbol-13-acetate (**8**), 12-(2-methylaminobenzoyl)-4-deoxyphorbaldehyde-13-acetae (**9**) were isolated from extract	Compound** 3 **(MIC, 3.12 *µ*g/ml) was the most active compound against H_37_Ra using MABA, followed by **7** (MIC, 12.5 *µ*g/ml) and then followed by **5**, **8,9** (MIC, 25 *µ*g/ml). Compound **1** showed moderate activity (MIC, 50 *µ*g/ml) while **2**, **4,6** showed weak activity (200, >200, >200 MIC, *µ*g/ml, respectively) [199].	Fever [200].

*Selaginella plana* (Desv. ex Poir.) Hieron.	Selaginellaceae	Paka merak	Whole plant: 80% methanol	In *in vitro* assay, the extract showed activity against H37Rv at 1600 *µ*g/ml MIC [57].	Coughing and asthma [57].

*Sesbania grandiflora* (L.) Poir.	Leguminosae	Geti	Fruit: 80% methanol	The extract exhibited inhibition against H37Rv strain at the MIC of 1600 *µ*g/ml [57].	Sore throat [201].
			Root: methanol/isovestitol (**1**), medicarpin (**2**), sativan (**3**), betulinic acid (**4**)	The methanol crude extract exhibited anti-TB activity against H37R*v* with MIC value of 625 *μ*g/ml. Isolated compounds 1-3 exhibited MIC of 50 *µ*g/ml while compound 4 showed activity at MIC of 100 *µ*g/ml [202].	

*Solanum spirale *Roxb.	Solanaceae	Pak dit	Fruit: water	Essential oil extracted showed anti-TB activity against H37Ra strain with MIC value of 50 *µ*g/ml [203].	Fevers and colds [203].

*Solanum torvum* Sw.	Solanaceae	Terung pipit	Fruit: 80% methanol	There was inhibition at the MIC of 1600 *μ*g/mL against H37Ra strain [57].	Cough [204].

*Spilanthes acmella *(L.) Murray	Asteraceae	Raan	Whole plant: chloroform methanol water	The chloroform, methanol, water extract of whole plant exhibited inhibition in MABA, against H37Ra strain at the MIC of 500, 1000, 1000 *µ*g/ml, respectively [50].	TB, cough, sore throats [205, 206].

*Spondias pinnata * (L.f.) Kurz	Anacardiaceae	Loloh cemcem	Chloroform, 80% ethanol.	The extracts were active against MDR strain of Lowenstein-Jensen medium with 100% inhibition at concentration of 100 mg/ml [207].	Chronic cough [207].

*Tabernaemontana coronaria* (L.) Willd.	Apocynaceae	Jasmine	Leaves: 80% methanol	At the MIC of 800 *µ*g/ml, the extract showed inhibition against H37Rv strain [57].	TB [57].

*Tiliacora triandra* (Colebr.) Diels	Menispermaceae	Ya-nang	Roots: tiliacorinine (**1**), 2′-nortiliacorinine (**2**), tiliacorine (**3**), 13′-bromo-tiliacorinine (**4**) were isolated from the extract	Compounds **1**, **2**, **3,4** demonstrated anti-TB activity against MDR-MTB strains with the MIC values ranging from 0.7 to 6.2 *µ*g/ml [208].	Fever [209].

*Tinospora crispa *(L.) Hook. F. & Thomson	Menispermaceae	Kheuah Khao Ho	Fruits and flowers: 90% ethanol	In microbroth dilution assay, the extract exhibited inhibition at MIC of 2.43 to 96.2 *µ*g/ml [56].	Coughs, asthma leprosy [210, 211].

*Trigonostemon reidioides* (Kurz) Craib	Euphorbiaceae	Lot Thanong	Root: n-hexane, EtOAc, MeOH/Compounds trigonoreidon A (**1**), trigonoreidon B (**2**), trigonoreidon C (**3**), trigonostemon C (**7**), spruceanol (**8**), trigonostemone (**9**), rediocide A (**10**), rediocide B (**11**), rediocide C (**12**), rediocide F (**13**), rediocide G (**14**) were isolated from the extract	Among the tested compounds, **12** and **14** were the two most active compounds against H37Ra strain with the MICs of 3.84 *µ*M, followed by **13**, with the MIC of 3.91 *µ*M. Compounds **11** and **10** were active and moderately active with the MICs of 7.86 and 15.72 *µ*M, respectively. Compounds **1**,** 2**,** 3**,** 7**,** 8, 9 **exhibited weak activity with MICs of 183.57, 168.71, 88.55, 83.79, 72.58, 38.30 *µ*M, respectively [212].	Asthma [212].

*Uvaria microcarpa *Champ. ex Benth.	Annonaceae	Phii Phouan	Fruits and flowers: 90% ethanol	The extract showed inhibition at MIC ranging from 43.2 to >100 *µ*g/ml against H37R*v* [56].	Nil.

*Uvaria rufa *Blume	Annonaceae	Mak Phii Phouan, Susung kalabaw	Fruits and flowers: 90% ethanol	The activity against H37R*v* strain was observed at the MIC ranging from 33.1 to >100 *µ*g/ml in MABA [56].	TB [213].
			Methanol, ethyl acetate, n-butanol Kaempferol (**1**), quercitrin (**2**) isolated from methanolic extract of leave	All three extracts showed inhibition in MIC value >128 *µ*g/ml in MABA. Mixture of **1** and **2** exhibited activities against H37Rv with MIC of 64 *µ*g/ml using microplate Alamar Blue assay [214].	

*Uvaria valderramensis * Cabuang, Exconde & Alejandro	Annonaceae	Usog	Leaves: dichloromethane and methanol/valderramenols A (**1**), grandiuvarone (**2**), andreticuline (**3**) isolated from the extract	Compound **1** showed better activity (10 *µ*g/ml) while **2** and **3** exhibited lesser activity (32 *µ*g/ml) against H37Rv using MABA [215].	

*Vitex trifolia *L.	Verbenaceae	Phi sua	90% ethanol	The extract showed inhibition at MIC value of 8.02 *µ*g/ml against H37R*v* [53].	Asthma, cough [53, 216].
			Fruits and flowers: 90% ethanol	In *in vitro* assays, 90% ethanolic fruit and flower extract exhibited activity against H37R*v* strain at the MIC ranging from 77.6 to >100 *µ*g/ml [56].	

*Voacanga globosa * Merr.	Apocynaceae	Bayag-usa	Leaves: dichloromethane and methane/Globospiramine (**1**) was isolated from the extract	**1** demonstrated potent anti-TB activity against H37Rv strain as demonstrated in MABA (MIC = 4 *µ*g/ml) and low-oxygen recovery assay (MIC = 5.2 *µ*g/ml) [217].	TB [218].

*Zingiber officinale* Roscoe	Zingiberaceae	Halia, Luya	Rhizome: 80% methanol/6-shogaol (**1**) and 6-gingerol (**2**) were isolated from the extract	In TEMA, the extract of methanol showed inhibition against H37R*v *strain at the MIC of 1600 *µ*g/ml [57]. At 100 *µ*g/ml, the methanolic extract of rhizomes showed 61% inhibition against H37Rv. The isolated compound **2** (MIC 33 *µ*g/ml) showed more activity than **1** (MIC 64 *µ*g/ml) [67].	Cough, asthma [219].

*Zingiber zerumbet *(L.) Roscoe ex Sm.	Zingiberaceae	Haeo dam	Rhizome: chloroform methanol water	In MABA, the chloroform, methanol, water extract of rhizome exhibited inhibition against H37Ra strain at the MIC of 125, 1000, 1000 *µ*g/ml, respectively [50].	Leprosy, cough, asthma, chest pain, loss of appetite [220, 221].

*Ziziphus mauritiana* Lam.	Rhamnaceae	Phut-sa	Root: mauritine M (**1**) and nummularines H (**2**), all isolated from ethanol extracts	Isolated compounds exhibited activities against H37Ra at the MIC 72.8 *µ*g/ml (**1**) and 4.5 *µ*g/ml (**2**) [222].	Asthma, bronchitis [223, 224].

Ziziphus oenoplia (L.) Mill.	Rhamnaceae		Root: hexane, ethyl acetate and methanol/Ziziphine N and Q were isolated from the extract	Ziziphine N and Q showed weak anti-TB activity against H37Ra strain with the same MIC value of 200 *µ*g/ml [225].	Asthma, fever [226].
